# Redox-dependent functional switching of plant proteins accompanying with their structural changes

**DOI:** 10.3389/fpls.2013.00277

**Published:** 2013-07-26

**Authors:** Yong Hun Chi, Seol Ki Paeng, Min Ji Kim, Gwang Yong Hwang, Sarah Mae B. Melencion, Hun Taek Oh, Sang Yeol Lee

**Affiliations:** Division of Applied Life Sciences, Gyeongsang National UniversityJinju, Korea

**Keywords:** external stress, molecular chaperone, multiple functions, redox proteins, structural and functional switching

## Abstract

Reactive oxygen species (ROS) can be generated during the course of normal aerobic metabolism or when an organism is exposed to a variety of stress conditions. It can cause a widespread damage to intracellular macromolecules and play a causal role in many degenerative diseases. Like other aerobic organisms plants are also equipped with a wide range of antioxidant redox proteins, such as superoxide dismutase, catalase, glutaredoxin, thioredoxin (Trx), Trx reductase, protein disulfide reductase, and other kinds of peroxidases that are usually significant in preventing harmful effects of ROS. To defend plant cells in response to stimuli, a part of redox proteins have shown to play multiple functions through the post-translational modification with a redox-dependent manner. For the alternative switching of their cellular functions, the redox proteins change their protein structures from low molecular weight to high molecular weight (HMW) protein complexes depending on the external stress. The HMW proteins are reported to act as molecular chaperone, which enable the plants to enhance their stress tolerance. In addition, some transcription factors and co-activators have function responding to environmental stresses by redox-dependent structural changes. This review describes the molecular mechanism and physiological significance of the redox proteins, transcription factors and co-activators to protect the plants from environmental stresses through the redox-dependent structural and functional switching of the plant redox proteins.

## INTRODUCTION

Plant cells produce various kinds of reactive oxygen species (ROS) from internal and external sources, such as hydrogen peroxide (H_2_O_2_), superoxide anions, and hydroxyl radicals. They can damage cellular components or act as important signal transduction molecules to trigger the cellular defense signaling cascades (Baier and Dietz, 2005; D’Autreaux and Toledano, 2007; Schwarzlander and Finkemeier, 2013). Thus, it is crucial for cells to detect the levels of ROS and activate defense signaling pathways (Moller and Sweetlove, 2010). To initiate cellular signaling cascades responding to a myriad of environmental signals, plants generate redox gradient across the plasma membrane, change metabolic activities, and trigger the inactivation of the oxidative burst-generating enzymes (Pignocchi and Foyer, 2003; Suzuki et al., 2012).

The alteration in steady-state level of ROS and subsequent changes of intracellular redox potential are important systems to regulate cellular signaling factors linking external stimuli with intracellular signal transduction pathway in response to stresses (Finkel, 2011). Plants are autotrophic organisms that are capable of undergoing photosynthesis by which they absorbed light energy that generate high electron and transport to chloroplasts, mitochondria, and peroxisomes along a cascade of redox components. During the reactions, ATP, NADPH, and other soluble reducing equivalents of ferredoxin (Fd), and thioredoxin (Trx) are generated (Schurmann and Buchanan, 2008). In addition, the thiol/disulfide state strongly regulates the light-dependent modulation of chloroplast enzyme activities (Scheibe, 1991). On the other hand, both the chloroplast and mitochondria originated as bacterial endosymbionts which retain a specialized genome and electron transport chains (ETCs; Murphy, 2009). The ETCs of plant mitochondria and chloroplast are major generator of ROS that contain flavin, metal centers, and quinones as their electron transport centers. Whereas, in the regulation of nuclear gene expression, plant peroxisomes controls intracellular ROS levels, plant photomorphogenesis, plant development, peroxisomal biogenesis, light signaling, and stress responses (Hu et al., 2002). In the moderate to high rates of photosynthesis, the peroxisome becomes the site of massive light-dependent generation of H_2_O_2._ They are multi-purpose organelles involved in fatty acid α-oxidation, β-oxidation of very long chain fatty acids, catabolism of purines, and biosynthesis of glycerolipids and bile acids. During the reactions, redox signals play important roles, since peroxisomes produce H_2_O_2_ at high rates through the reactions of β-oxidation of long chain fatty acid and glycolate oxidation (Jimenez et al., 1997).

**FIGURE 1:**
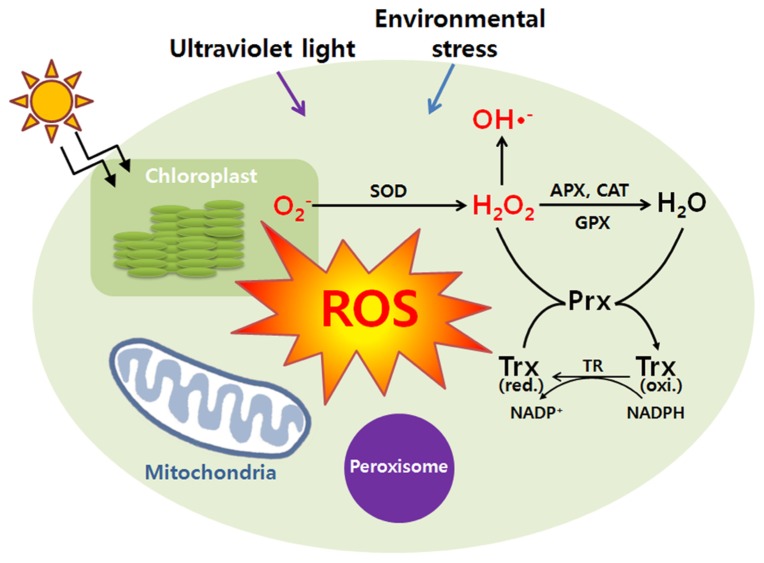
** Cellular responses against reactive oxygen species.** During a variety of stress conditions (UV, environmental stress, etc), reactive oxygen species (ROS) can be generated. At the start of the reaction, a primary ROS which is superoxide anion (O^2^^-^) can be formed by the one electron reduction of molecular oxygen. The superoxide anion (O^2^^-^) is dismutated by superoxide dismutase (SOD) to hydrogen peroxide (H_2_O_2_) which is detoxified by catalase (CAT), ascorbate peroxidase (APX), glutathione peroxidase (GPX), and peroxiredoxin (Prx). Once the superoxide anion (O^2^^-^) is formed in the presence of H_2_O_2_, it becomes inevitable. Further reactions may lead to the formation of hydroxyl radicals (HO^·^). Antioxidant proteins protected the damages of ROS leaking from peroxisomes. H_2_O_2_ can easily permeate the peroxisomal membrane and play an important role in protection of cells from ROS damages. And various ROS transmitted to the mitochondrion play a role in the adaptive response of mitochondrial redox state, especially for the reduction state of respiratory pathways. The redox signals will be transmitted to the nucleus to regulate plant growth and developments. Trx represents thioredoxin.

To integrate ROS production from major sources of plant cells, they produce large quantities of redox-materials to buffer the intracellular redox changes by expressing many kinds of soluble hydrophilic antioxidants, ascorbate, glutathione, and redox-regulating proteins including Trx, glutaredoxin (Grx), protein disulfide reductase (PDI), etc. (**Figure 1**). These compounds are important factors to determine the lifetime of H_2_O_2_ and redox potential of plant cells (Noctor and Foyer, 1998; Foyer and Noctor, 2013). Changes in the redox state of these components regulate the expression of both plastome- and nuclear-encoded proteins. Furthermore, the redox information co-ordinates the gene expression located in the compartments of chloroplasts, mitochondria, and nucleus (Allen and Pfannschmidt, 2000). Redox signals also leave the chloroplast and mitochondria to provide a decisive input into transcriptional control of the plant nucleus. Thus, redox signaling plays a key role in the coordination between the cytoplasmic genomes in the chloroplast and mitochondria and the nucleus functioning within the global network of whole plants and it may also provide evidence of many cellular proteins and enzyme activities that should be precisely regulated in response to plant growth, development, and differentiation.

## STRUCTURAL AND FUNCTIONAL REGULATION OF REDOX-DEPENDENT TRANSCRIPTIONAL FACTOR AND CO-ACTIVATORS

**FIGURE 2:**
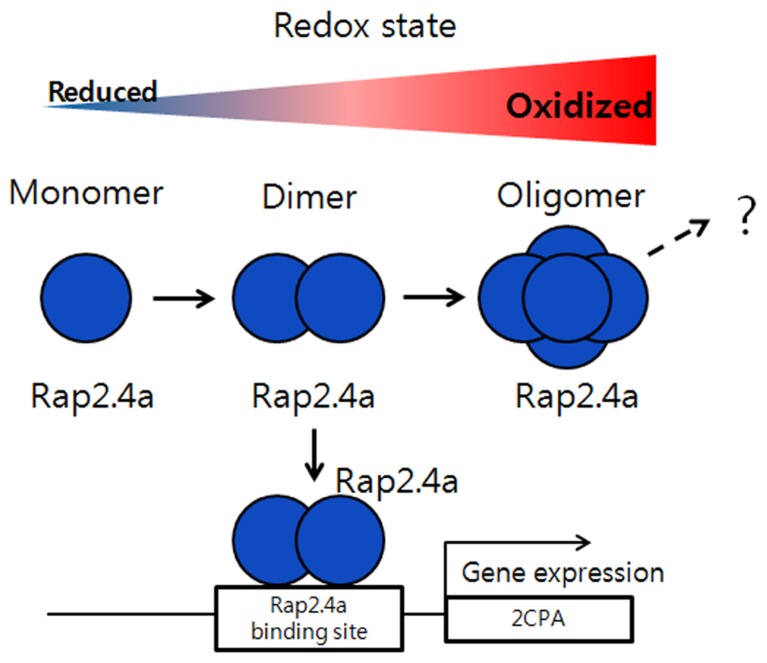
** Regulation of gene expression by Rap2.4a through redox changes.** Under oxidizing conditions, Rap2.4a changes its structure from monomer, dimer to oligomer. The homodimeric structure of Rap2.4a can bind to the DNA promoter of antioxidant like 2-Cys peroxiredoxin and plays as transcription factor to regulate its gene expression. If the redox balance changes to more oxidized condition, the expression level of Rap2.4a is increased. But, during severe oxidative conditions, Rap2.4a expression is decreased and lost its transcriptional activity due to oligomerization. This model is modified from the reference of Shaikhali et al. (2008).

Reactive oxygen species can cause widespread damage to biological macromolecules (Halliwell and Gutteridge, 1990; Alvarez et al., 1998). In order to protect themselves from oxidative stress and ROS-mediated protein unfolding and aggregation, plant cells are equipped with a wide range of antioxidant proteins, including superoxide dismutase, catalase, peroxidases, and diverse forms of molecular chaperones, small heat shock proteins (Hendrick and Hartl, 1993; Dietz, 2003). Furthermore, several moon-light proteins have multiple functions responding to foreign stresses, such as ROS, heat shock, and pathogen attacks, accompanying with their structural changes (Lee et al., 2009; Park et al., 2009; Chae et al., 2013). In this review, we will introduce several representative examples of the proteins which switch their protein structures and functions with a redox-dependent manner in response to external stresses. Redox-regulation is a fine-tuning mechanism for the transcription of plant genomes in nucleus, chloroplast, and mitochondria to co-ordinate with plant development and differentiation along with environmental parameters (Baier and Dietz, 2005; Piippo et al., 2006; Schwarzlander and Finkemeier, 2013). There are several redox sensitive transcriptional factors whose activities are relied on redox- and structure-dependent manner (Tron et al., 2002; Heine et al., 2004; Serpa et al., 2007; Shaikhali et al., 2008, 2012). Among them, the redox-dependent transcriptional factor, Rap2.4a, was isolated and studied as an efficient redox-sensor and transducer of redox status of cells to nucleus to control transcriptional activity of chloroplast antioxidant proteins (Shaikhali et al., 2008). The protein was cloned from a yeast-one-hybrid screening with a *cis*-regulatory element of 2-Cys peroxiredoxin (Prx)-A gene (2CPA) is grouped into activator protein 2 (AP2)-type transcription factor (Srivastava et al., 2009). Rap2.4a transcriptional activity is regulated by dithiol/disulfide transition of regulatory cysteinyl residues (Shaikhali et al., 2008). During the processes, the protein changes its quaternary structures according to redox status. Under oxidizing conditions, Rap2.4a switches its structure from monomer and dimer to polymers, respectively. And the dimeric form of Rap2.4a plays a critical role as a transcription factor to stimulate nuclear gene expression of the photosynthetic chloroplast enzymes (Shaikhali et al., 2008). Thus, the homodimeric structure of Rap2.4a formed by intermolecular disulfide bond is the active form necessary for DNA binding and transcriptional activity. Also the oxidation of the dimer by H_2_O_2_ or reduction by dithiothreitol (DTT) significantly changes its protein structures to make homopolymer and monomer and reduces its DNA binding and transcriptional activity (**Figure 2**; Shaikhali et al., 2008, 2012).

Another example of transcription factor regulating its activity by structural changes is given in basic leucine zipper (bZIP) transcription factor in *Arabidopsis*, AtbZIP16 (Shaikhali et al., 2012). This protein belongs to the G-group of *Arabidopsis* bZIP type transcription factors whose promoters are responsive to a variety of environmental stimuli (Kleine et al., 2007). bZip16 has a conserved Cys residues that plays a critical role in redox regulation of the target gene expression, which is proven by the transgenic *Arabidopsis* overexpressing the Cys mutated variant of bZIP16 (Shaikhali et al., 2012). Multiple protein bands are detected corresponding to monomer, dimer, and oligomer forms of AtbZIP16 in the non-reducing conditions (Shaikhali et al., 2012). And the oligomeric and dimeric forms are reduced by increasing DTT concentrations, which results in a complete conversion to a monomer. In contrast, increasing the concentration of H_2_O_2_ produces a gradual loss of the small molecular forms, which suggests that H_2_O_2_ induces the formation of very high molecular mass complexes in a reversible way. The high molecular mass oligomers can be reversibly dissociated by the treatment of reducing agents, such as DTT. Only DTT-mediated dissociated AtbZIP16 can bind to DNA promoter and functions as a transcription factor (Shaikhali et al., 2012). The mutated Cys variant form of bZIP16 has an important physiological significance of its conserved Cys residue in redox regulation of gene expression. Based on these data, the redox-dependent structural changes is highly important to modulate the activity of these transcription factors in response to environmental signals.

**FIGURE 3:**
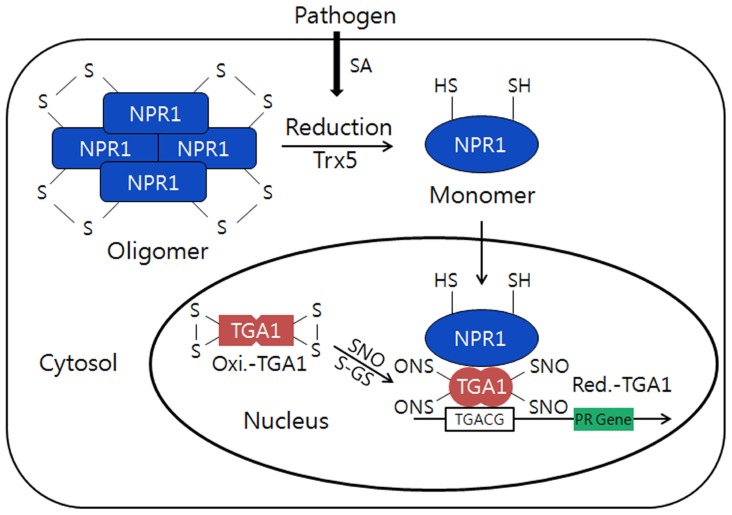
** Conformational switching from the oligomers to a monomer of NPR1 by redox changes in plants.** Under normal conditions, NPR1forms an inactive oligomer structure and is oxidized by intermolecular disulfide bonds in the cytosol. However, during pathogen attack, the NPR1 changes its structure from inactive to active form of monomer through the reduction of the intermolecular bridges by h5-type thioredoxin (AtTrx-h5). Then, NPR1 will be translocated to the nucleus and interacts withTGA1transcription factor that can induce PR gene expression. TGA1 in normal condition is inactive and oxidized form with intramolecular disulfide bonds in the nucleus. However, in stress conditionTGA1 will be reduced and interact with NPR1 and can enhance its DNA binding activity through *S*-nitrosylation (SNO) and *S*-glutathionylation (*S*-GS) by GSNO and glutathione (GSH/GSSG). This model is modified from the reference of Moore et al. (2011).

A typical redox protein regulating its protein structure and functions against external biotic stress can also be found from the transcriptional co-activator, NPR1 (NON-EXPRESSOR OF PR1; Mou et al., 2003; Tada et al., 2008). The gene was originally cloned from *Arabidopsis* mutant screening to identify the loss of ability to respond to inducers of systemic acquired resistance (SAR) such as salicylic acid (SA), and designated *npr*-1, *nim*-1, and *sai*1, simultaneously. The NPR1 having no DNA-binding domain acts as a co-activator by specifically associated with the specific DNA-binding transcription factor, TGACG sequence-specific binding proteins (TGAs), and plays a critical role in plant pathogen resistance (Grant and Lamb, 2006; Spoel et al., 2009; Maier et al., 2011). NPR1 containing several conserved Cys residues controls the expression of over 2,200 defense response genes in *Arabidopsis *and has stable structure that forms a high molecular weight (HMW) oligomer, which makes the protein confine at the plant cytoplasm (Mou et al., 2003; Wang et al., 2006). The *npr1 *mutants neither accumulate pathogen-related (PR) proteins in response to SA nor exhibit resistance against pathogen infection, such as downy mildew. The *NPR1* gene contains a BTB (BR-C, ttk and bab)/POZ (Pox virus and Zinc finger) domain at its N-terminus which facilitates dimerization of NPR1 and ankyrin repeats in the central region that is involved in the interaction with TGA sub-family of bZIP transcription factors (Stogios et al., 2005; Boyle et al., 2009). Under the normal conditions, NPR1 exists as an inactive form of oligomers that are associated by intermolecular disulfide bonds in the cytosol (**Figure 3**). However, the redox changes triggered by SA, a phytohormone produced in response to pathogen attack, induce the release of NPR1 from the oligomers to an active form of monomer by reduction of the intermolecular disulfide bridges (Dong, 2004; Moore et al., 2011; Astier et al., 2012). Then, the NPR1 monomer translocates from the cytosol to nucleus and stimulates the transcriptional activation of defense-related genes through the binding with transcription factors belonging to TGA sub-family (Dong, 2004; Moore et al., 2011; Astier et al., 2012). Particularly, the *S*-nitrosylation of Cys^156^ has been shown to trigger conformational changes of NPR1 facilitating disulfide linkage between NPR1 monomers to form inactive homopolymers. During the redox-mediated switching of its function and structures from the inactive oligomers located in cytosol to the active monomeric NPR1 that will be translocated to the nucleus, Trx-h5 plays an important role in the NPR1 dissociate ion and activation. NPR1 may be the most important gene for the preparation of broad spectrum disease-resistant transgenic crops (Cao et al., 1994; Delaney et al., 1995; Shah et al., 1997).

## FUNCTIONAL SWITCHING OF REDOX PROTEINS ACCOMPANYING WITH THEIR REDOX-DEPENDENT STRUCTURAL CHANGES

Thioredoxin is a general disulfide oxidoreductase and a ubiquitous redox protein with a single disulfide bridge in all organisms. The function of Trx is involved in numerous redox-dependent cellular processes, such as activation of ribonucleotide reductase, photosynthetic activity of plant cells, modulation of transcription factors, and promotion of a variety of diseases (Aslund and Beckwith, 1999; Balmer et al., 2003; Ravi et al., 2005). Trxs also control several redox-independent cellular reactions including an assembly of T7 DNA polymerase complex and formation of filamentous phage (Feng et al., 1997; Hamdan et al., 2005). The proteins belonging to Trx group share high amino acid sequence similarity and contain a common structural motif, the Trx-fold. The Trx-fold comprises approximately 80 amino acid residues with a central core of five β-strands that are enclosed by four α-helices and two hydrophobic zones (Katti et al., 1990). The interesting point we want to focus in this review is that some of redox proteins harboring the Trx-fold have been shown to behave as molecular chaperones with their endogenous reductase function. The proteins include Trx-like domain containing protein (TDX), protein disulfide isomerase, and 2-Cys-Prxs (Quan et al., 1995; Jang et al., 2004), etc. To be a molecular chaperone, it should interact with target substrates and switch its protein structures in response to external stresses with a reversible fashion (Jang et al., 2004; Lee et al., 2009; Park et al., 2009). The Trx-fold containing proteins interact with substrate proteins through their hydrophobic surfaces around their active sites and reversibly change the protein structures as the following examples.

Among the various kinds of Trx isoforms, a specific type of *Arabidopsis* Trx in cytosol, AtTrx-h3, forms various protein structures ranging from low molecular weight (LMW) protein species to HMW homo-complexes, which are verified by size exclusion chromatography, native-PAGE gel, and electro-microscopic analyses (Park et al., 2009). The AtTrx-h3 performs dual functions, acting as a disulfide reductase and as a molecular chaperone, which are closely associated with its differently sized multiple protein structures. The disulfide reductase function is observed predominantly in the LMW forms, whereas the chaperone function predominates in the HMW complexes. The multimeric structures of AtTrx-h3 are regulated by redox status. The reduction of AtTrx-h3 by DTT changes the HMW structures of AtTrx-h3 into LMW protein species, and subsequent treatment of hydrogen peroxide (H_2_O_2_) after removal of DTT almost restores the HMW structures of AtTrx-h3. Particularly, the AtTrx-h3 polymeric structures are associated not only by the forces of hydrophobic interaction but also by the redox-dependent disulfide bonds. Two active cysteine residues in AtTrx-h3 are required for disulfide reductase activity, but not for chaperone function. Thus, the active site mutant protein, C39/42S-AtTrx-h3, is not able to reduce disulfide bonds of substrate at all, but has nearly the same chaperone activity as that of native AtTrx-h3 protein. The transgenic lines overexpressing native AtTrx-h3 or C39/42S (DM) mutant AtTrx-h3 having only the chaperone function exhibit enhanced heat shock tolerance compared to wild-type plants. From the results, it can be concluded that the AtTrx-h3 plays a pivotal role in the protection of plant cells from external stresses through its chaperone function (Park et al., 2009).

In addition to the At-Trx-h3, the plant-specific Trx-like protein containing 3 tetratricopeptide repeat (TPR) domains and a Trx motif which is designated AtTDX has a highly heat-stable property. The TPR units in AtTDX are particularly important for protein–protein interaction and formation of multi-protein complexes (Blatch and Lassle, 1999), which are characteristic properties of molecular chaperones. AtTDX has diverse protein structures consisting of monomer, dimer, oligomer, and HMW complexes. The protein also displays multiple functions, acting as a disulfide reductase, foldase chaperone, and holdase chaperone. In particular, the functions of AtTDX are closely associated with its oligomerization status. Like the AtTrx-h3, multimerization of AtTDX enhances its holdase chaperone activity, whereas dissociation promotes its disulfide reductase and foldase chaperone functions. However, when the TPR domains of AtTDX is removed, the truncated protein shows a significant enhancement of its disulfide reductase activity but results in a complete loss of the holdase chaperone function of AtTrx-h3 (Lee et al., 2009). The result suggests the TPR domains of AtTDX completely block the active site of Trx motif and play a critical role in promoting the holdase chaperone function. Moreover, the Cys mutant proteins (C304S, C307S, and C304/307S) of AtTDX do not exhibit disulfide reductase activity but display a similar activity of holdase chaperone function as native AtTDX. The results suggest that the active site Cys residues critically contribute to the reductase function but not the chaperone function. For the regulation of its multiple functions, protein structure of AtTDX is varied against external conditions. The oligomerization status of AtTDX is reversibly controlled by heat shock and ROS concentrations, which cause a transition from LMW to HMW complexes with a concomitant functional switching from a disulfide reductase and foldase chaperone to a holdase chaperone. It is generally known that the chaperone function contributes resistance to cells against external stresses. Thus, when the heat-stressed *Arabidopsis *of the WT, AtTDX overexpression lines, AtTDX suppression lines, and Cys-mutant (C304/307S) AtTDX overexpression lines having only the holdase chaperone function are returned to their optimal temperature, the transgenic lines overexpressing the native form and C304/307S mutant form of AtTDX recover during the post-stress recovery period. In contrast, AtTDX suppression lines of *Arabidopsis* show a highly sensitive phenotype against heat shock. The results can conclude that the holdase chaperone function of AtTDX plays a major role in the protection of *Arabidopsis* from heat stress during the heat shock and/or recovery period (Lee et al., 2009).

Another redox protein sharing a similar regulation mode with AtTrx-h3 and AtTDX proteins can be found from the C-type of NADPH-dependent Trx reductase (NTRC), which is a new member of the plant-specific NADPH-dependent Trx reductase (NTR) family. During the early evolution of chloroplasts, the NTRC appears to be originated from cyanobacteria by the transfer of this gene into the plant genome. The protein contains an N-terminal Trx reductase (TR) domain and a Trx domain at the C-terminus. The functional role of this fusion of domains in NTRC has been verified as an efficient electron donor to 2Cys-Prx (Moon et al., 2006; Perez-Ruiz et al., 2006). Particularly, *Arabidopsis* NTRC shows enzymatic activity characteristic for each of its separate domains and in a combination of the TR and Trx domains (Moon et al., 2006). The disulfide reductase function of NTRC is coupled with the reducing power, NADPH, which is produced by photosynthetic electron transport systems in the light conditions. The knockout mutant of *Arabidopsis* NTRC exhibits growth inhibition under stress conditions and shows reduced auxin levels (Serrato et al., 2004; Lepisto et al., 2009). In the letter case, the mutant phenotypes are restored by supplementing growth medium with tryptophan and phenylalanine. Interestingly, it has been reported that the protein structures of NTRC have various oligomeric conformations in other species like rice, barrel medic, and barley (Alkhalfioui et al., 2007; Perez-Ruiz and Cejudo, 2009; Wulff et al., 2011). That is, NTRC assembles into homopolymeric structures of varying complexity with functions as a disulfide reductase, a foldase chaperone, and as a holdase chaperone. The multiple functions of NTRC are also associated with its protein structures. Complexes of HMW show stronger activity as a holdase chaperone, whereas the LMW species exhibit weaker holdase chaperone activity with stronger disulfide reductase and foldase chaperone activities (**Table 1**). Heat shock converts LMW proteins into HMW complexes and gradually increases the holdase chaperone function of NTRC. Upon the heat shock treatment, NTRC results in a decrease in its disulfide reductase and foldase chaperone activities. In conclusion, heat shock-mediated oligomeric changes of NTRC are closely associated with a change in its functional switching from a disulfide reductase to a molecular chaperone.

**Table 1:** Structural and functional switching of NTRC in* Arabidopsis thaliana *in response to redox state.

NTRC	Redox state	
	Reduction	Oxidation
Protein structures	Low molecular weight species	High molecular weight complexes
	•Monomer	•Oligomer
	•Dimer	
Functions	Disulfide reductase Foldase chaperone	Holdase chaperone

## CONCLUSION AND PERSPECTIVE

To cope with external stresses, plants regulate their protein functions by employing a number of efficient regulation strategies, such as phosphorylation/dephosphorylation, covalent modification, proteolytic degradation or activation, interacting with partner proteins, and so on. However, at recent, the post-translational modification is identified as one of the most important, rapid and precise methods to respond eukaryotic cells against environmental stresses. The redox-dependent functional switching is a typical scheme for the plant defense systems. Particularly, the functional shift of the redox proteins is accompanied with their structural changes in response to redox changes. In this review, we introduce several examples of the redox proteins to respond environmental circumstances. However, besides the redox proteins in eukaryotes, many redox-independent proteins showing similar regulation pattern with the proteins have also been identified from various sources, such as plant phosphodiesterase, sodium/proton antiporter (NHX), salt overly sensitive 1 (SOS1), mammalian nuclear factor kappa beta (mammalian NF-kB) and AP1, yeast Yap1, bacterial OxyR/S, and so on (Zheng et al., 1998; Toone et al., 2001; Kim et al., 2002, 2012; Hess et al., 2004; D’Autreaux and Toledano, 2007; Salminen et al., 2008; Rodriguez-Rosales et al., 2009; Ji et al., 2013). Thus, these elaborate functional regulation mode allows higher eukaryotic organisms to precisely respond to external stresses and to survive from the harsh and changeable environmental conditions. This review provides valuable insights into how plants can respond to the rapid changes of redox potential induced by biotic/abiotic stresses at the molecular level.

## Conflict of Interest Statement

The authors declare that the research was conducted in the absence of any commercial or financial relationships that could be construed as a potential conflict of interest.

## References

[B1] Alkhalfioui F. Renard M. Montrichard F. (2007). Unique properties of NADP-thioredoxin reductase C in legumes. *J. Exp. Bot.* 58 969–978 10.1093/jxb/erl248 17185738

[B2] Allen J. F. Pfannschmidt T. (2000). Balancing the two photosystems: photosynthetic electron transfer governs transcription of reaction centre genes in chloroplasts. *Philos. Trans. R. Soc. Lond. B Biol. Sci.* 355 1351–1359 10.1098/rstb.2000.0697 11127990 PMC1692884

[B3] Alvarez M. E. Pennell R. I. Meijer P. J. Ishikawa A. Dixon R. A. Lamb C. (1998). Reactive oxygen intermediates mediate a systemic signal network in the establishment of plant immunity. *Cell* 92 773–784 10.1016/S0092-8674(00)81405-1 9529253

[B4] Aslund F. Beckwith J. (1999). Bridge over troubled waters: sensing stress by disulfide bond formation. *Cell* 96 751–753 10.1016/S0092-8674(00)80584-X 10102262

[B5] Astier J. Kulik A. Koen E. Besson-Bard A. Bourque S. Jeandroz S. (2012). Protein *S*-nitrosylation: what’s going on in plants? *Free Radic. Biol. Med.* 53 1101–1110 10.1016/j.freeradbiomed.2012.06.032 22750205

[B6] Baier M. Dietz K. J. (2005). Chloroplasts as source and target of cellular redox regulation: a discussion on chloroplast redox signals in the context of plant physiology. *J. Exp. Bot.* 56 1449–1462 10.1093/jxb/eri161 15863449

[B7] Balmer Y. Koller A. Del Val G. Manieri W. Schurmann P. Buchanan B. B. (2003). Proteomics gives insight into the regulatory function of chloroplast thioredoxins. *Proc. Natl. Acad. Sci. U.S.A.* 100 370–375 10.1073/pnas.232703799 12509500 PMC140980

[B8] Blatch G. L. Lassle M. (1999). The tetratricopeptide repeat: a structural motif mediating protein–protein interactions. *Bioessays* 21 932–939 10.1002/(SICI)1521-1878(199911)21:11 10517866

[B9] Boyle P. Le Su E. Rochon A. Shearer H. L. Murmu J. Chu J. Y. (2009). The BTB/POZ domain of the *Arabidopsis *disease resistance protein NPR1 interacts with the repression domain of TGA2 to negate its function. *Plant Cell* 21 3700–3713 10.1105/tpc.109.069971 19915088 PMC2798319

[B10] Cao H. Bowling S. A. Gordon A. S. Dong X. (1994). Characterization of an *Arabidopsis* mutant that is nonresponsive to inducers of systemic acquired resistance. *Plant Cell* 6 1583–1592 10.1105/tpc.6.11.1583 12244227 PMC160545

[B11] Chae H. B. Moon J. C. Shin M. R. Chi Y. H. Jung Y. J. Lee S. Y. (2013). Thioredoxin reductase type C (NTRC) orchestrates enhanced thermotolerance to *Arabidopsis* by its redox-dependent holdase chaperone function. *Mol. Plant* 6 323–336 10.1093/mp/sss105 23024205

[B12] D’Autreaux B. Toledano M. B. (2007). ROS as signalling molecules: mechanisms that generate specificity in ROS homeostasis. *Nat. Rev. Mol. Cell Biol.* 8 813–824 10.1038/nrm2256 17848967

[B13] Delaney T. P. Friedrich L. Ryals J. A. (1995). *Arabidopsis* signal transduction mutant defective in chemically and biologically induced disease resistance. *Proc. Natl. Acad. Sci. U.S.A.* 92 6602–6606 10.1073/pnas.92.14.6602 11607555 PMC41566

[B14] Dietz K. J. (2003). Redox control, redox signaling, and redox homeostasis in plant cells. *Int. Rev. Cytol.* 228 141–193 10.1016/S0074-7696(03)28004-9 14667044

[B15] Dong X. (2004). NPR1, all things considered. *Curr. Opin. Plant Biol.* 7 547–552 10.1016/j.pbi.2004.07.005 15337097

[B16] Feng J. N. Russel M. Model P. (1997). A permeabilized cell system that assembles filamentous bacteriophage. *Proc. Natl. Acad. Sci. U.S.A.* 94 4068–4073 10.1073/pnas.94.8.4068 9108106 PMC20569

[B17] Finkel T. (2011). Signal transduction by reactive oxygen species. *J. Cell Biol.* 194 7–15 10.1083/jcb.201102095 21746850 PMC3135394

[B18] Foyer C. H. Noctor G. (2013). Redox signaling in plants. *Antioxid. Redox Signal.* 18 2087–2090 10.1089/ars.2013.5278 23442120

[B19] Grant M. Lamb C. (2006). Systemic immunity. *Curr. Opin. Plant Biol.* 9 414–420 10.1016/j.pbi.2006.05.013 16753329

[B20] Halliwell B. Gutteridge J. M. (1990). Role of free radicals and catalytic metal ions in human disease: an overview. *Methods Enzymol.* 186 1–85 10.1016/0076-6879(90)86093-B 2172697

[B21] Hamdan S. M. Marintcheva B. Cook T. Lee S. J. Tabor S. Richardson C. C. (2005). A unique loop in T7 DNA polymerase mediates the binding of helicase-primase, DNA binding protein, and processivity factor. *Proc. Natl. Acad. Sci. U.S.A.* 102 5096–5101 10.1073/pnas.0501637102 15795374 PMC556000

[B22] Heine G. F. Hernandez J. M. Grotewold E. (2004). Two cysteines in plant R2R3 MYB domains participate in REDOX-dependent DNA binding. *J. Biol. Chem.* 279 37878–37885 10.1074/jbc.M405166200 15237103

[B23] Hendrick J. P. Hartl F. U. (1993). Molecular chaperone functions of heat-shock proteins. *Annu. Rev. Biochem.* 62 349–384 10.1146/annurev.bi.62.070193.002025 8102520

[B24] Hess J. Angel P. Schorpp-Kistner M. (2004). AP-1 subunits: quarrel and harmony among siblings. *J. Cell Sci.* 117 5965–5973 10.1242/jcs.01589 15564374

[B25] Hu J. Aguirre M. Peto C. Alonso J. Ecker J. Chory J. (2002). A role for peroxisomes in photomorphogenesis and development of *Arabidopsis*. *Science* 297 405–409 10.1126/science.1073633 12130786

[B26] Jang H. H. Lee K. O. Chi Y. H. Jung B. G. Park S. K. Park J. H. (2004). Two enzymes in one; two yeast peroxiredoxins display oxidative stress-dependent switching from a peroxidase to a molecular chaperone function. *Cell* 117 625–635 10.1016/j.cell.2004.05.002 15163410

[B27] Ji H. Pardo J. M. Batelli G. Van Oosten M. J. Bressan R. A. Li X. (2013). The Salt Overly Sensitive (SOS) pathway: established and emerging roles. *Mol. Plant* 6 275–286 10.1093/mp/sst017 23355543

[B28] Jimenez A. Hernandez J. A. Del Rio L. A. Sevilla F. (1997). Evidence for the presence of the ascorbate-glutathione cycle in mitochondria and peroxisomes of pea leaves. *Plant Physiol.* 114 275–284 10.1104/pp.114.1.275 12223704 PMC158303

[B29] Katti S. K. Lemaster D. M. Eklund H. (1990). Crystal structure of thioredoxin from *Escherichia coli* at 1.68 A resolution. *J. Mol. Biol.* 212 167–184 10.1016/0022-2836(90)90313-B 2181145

[B30] Kim H. Na S. H. Lee S. Y. Jeong Y. M. Hwang H. J. Hur J. Y. (2012). Structure-function studies of a plant tyrosyl-DNA phosphodiesterase provide novel insights into DNA repair mechanisms of *Arabidopsis thaliana*. *Biochem. J.* 443 49–56 10.1042/BJ20111308 22214184 PMC3304491

[B31] Kim S. O. Merchant K. Nudelman R. Beyer W. F. Jr. Keng T. Deangelo J. (2002). OxyR: a molecular code for redox-related signaling. *Cell* 109 383–396 10.1016/S0092-8674(02)00723-7 12015987

[B32] Kleine T. Kindgren P. Benedict C. Hendrickson L. Strand A. (2007). Genome-wide gene expression analysis reveals a critical role for CRYPTOCHROME1 in the response of *Arabidopsis* to high irradiance. *Plant Physiol.* 144 1391–1406 10.1104/pp.107.098293 17478635 PMC1914119

[B33] Lee J. R. Lee S. S. Jang H. H. Lee Y. M. Park J. H. Park S. C. (2009). Heat-shock dependent oligomeric status alters the function of a plant-specific thioredoxin-like protein, AtTDX. *Proc. Natl. Acad. Sci. U.S.A.* 106 5978–5983 10.1073/pnas.0811231106 19293385 PMC2667072

[B34] Lepisto A. Kangasjarvi S. Luomala E. M. Brader G. Sipari N. Keranen M. (2009). Chloroplast NADPH-thioredoxin reductase interacts with photoperiodic development in *Arabidopsis*. *Plant Physiol.* 149 1261–1276 10.1104/pp.108.133777 19151130 PMC2649390

[B35] Maier F. Zwicker S. Huckelhoven A. Meissner M. Funk J. Pfitzner A. J. (2011). NONEXPRESSOR OF PATHOGENESIS-RELATED PROTEINS1 (NPR1) and some NPR1-related proteins are sensitive to salicylic acid. *Mol. Plant Pathol.* 12 73–91 10.1111/j.1364-3703.2010.00653.x 21118350 PMC6640455

[B36] Moller I. M. Sweetlove L. J. (2010). ROS signalling–specificity is required. *Trends Plant Sci.* 15 370–374 10.1016/j.tplants.2010.04.008 20605736

[B37] Moon J. C. Jang H. H. Chae H. B. Lee J. R. Lee S. Y. Jung Y. J. (2006). The C-type *Arabidopsis* thioredoxin reductase ANTR-C acts as an electron donor to 2-Cys peroxiredoxins in chloroplasts. *Biochem. Biophys. Res. Commun.* 348 478–484 10.1016/j.bbrc.2006.07.088 16884685

[B38] Moore J. W. Loake G. J. Spoel S. H. (2011). Transcription dynamics in plant immunity. *Plant Cell* 23 2809–2820 10.1105/tpc.111.087346 21841124 PMC3180793

[B39] Mou Z. Fan W. Dong X. (2003). Inducers of plant systemic acquired resistance regulate NPR1 function through redox changes. *Cell* 113 935–944 10.1016/S0092-8674(03)00429-X 12837250

[B40] Murphy M. P. (2009). How mitochondria produce reactive oxygen species. *Biochem. J.* 417 1–13 10.1042/BJ20081386 19061483 PMC2605959

[B41] Noctor G. Foyer C. H. (1998). ASCORBATE AND GLUTATHIONE: keeping active oxygen under control. *Annu. Rev. Plant Physiol. Plant Mol. Biol.* 49 249–279 10.1146/annurev.arplant.49.1.249 15012235

[B42] Park S. K. Jung Y. J. Lee J. R. Lee Y. M. Jang H. H. Lee S. S. (2009). Heat-shock and redox-dependent functional switching of an h-type *Arabidopsis* thioredoxin from a disulfide reductase to a molecular chaperone. *Plant Physiol.* 150 552–561 10.1104/pp.109.135426 19339505 PMC2689952

[B43] Perez-Ruiz J. M. Cejudo F. J. (2009). A proposed reaction mechanism for rice NADPH thioredoxin reductase C, an enzyme with protein disulfide reductase activity. *FEBS Lett.* 583 1399–1402 10.1016/j.febslet.2009.03.067 19345687

[B44] Perez-Ruiz J. M. Spinola M. C. Kirchsteiger K. Moreno J. Sahrawy M. Cejudo F. J. (2006). Rice NTRC is a high-efficiency redox system for chloroplast protection against oxidative damage. *Plant Cell* 18 2356–2368 10.1105/tpc.106.041541 16891402 PMC1560923

[B45] Pignocchi C. Foyer C. H. (2003). Apoplastic ascorbate metabolism and its role in the regulation of cell signalling. *Curr. Opin. Plant Biol.* 6 379–389 10.1016/S1369-5266(03)00069-4 12873534

[B46] Piippo M. Allahverdiyeva Y. Paakkarinen V. Suoranta U. M. Battchikova N. Aro E. M. (2006). Chloroplast-mediated regulation of nuclear genes in *Arabidopsis thaliana* in the absence of light stress. *Physiol. Genomics* 25 142–152 10.1152/physiolgenomics.00256.2005 16403842

[B47] Quan H. Fan G. Wang C. C. (1995). Independence of the chaperone activity of protein disulfide isomerase from its thioredoxin-like active site. *J. Biol. Chem.* 270 17078–17080 10.1074/jbc.270.29.17078 7615500

[B48] Ravi D. Muniyappa H. Das K. C. (2005). Endogenous thioredoxin is required for redox cycling of anthracyclines and p53-dependent apoptosis in cancer cells. *J. Biol. Chem.* 280 40084–40096 10.1074/jbc.M507192200 16159878

[B49] Rodriguez-Rosales M. P. Galvez F. J. Huertas R. Aranda M. N. Baghour M. Cagnac O. (2009). Plant NHX cation/proton antiporters. *Plant Signal. Behav.* 4 265–276 10.4161/psb.4.4.7919 19794841 PMC2664485

[B50] Salminen A. Huuskonen J. Ojala J. Kauppinen A. Kaarniranta K. Suuronen T. (2008). Activation of innate immunity system during aging: NF-kB signaling is the molecular culprit of inflamm-aging. *Ageing Res. Rev.* 7 83–105 10.1016/j.arr.2007.09.002 17964225

[B51] Scheibe R. (1991). Redox-modulation of chloroplast enzymes : a common principle for individual control. *Plant Physiol.* 96 1–3 10.1104/pp.96.1.1 16668135 PMC1080704

[B52] Schurmann P. Buchanan B. B. (2008). The ferredoxin/thioredoxin system of oxygenic photosynthesis. *Antioxid. Redox Signal.* 10 1235–1274 10.1089/ars.2007.1931 18377232

[B53] Schwarzlander M. Finkemeier I. (2013). Mitochondrial energy and redox signaling in plants. *Antioxid. Redox Signal.* 18 2122–2144 10.1089/ars.2012.5104 23234467 PMC3698670

[B54] Serpa V. Vernal J. Lamattina L. Grotewold E. Cassia R. Terenzi H. (2007). Inhibition of AtMYB2 DNA-binding by nitric oxide involves cysteine *S*-nitrosylation. *Biochem. Biophys. Res. Commun.* 361 1048–1053 10.1016/j.bbrc.2007.07.133 17686455

[B55] Serrato A. J. Perez-Ruiz J. M. Spinola M. C. Cejudo F. J. (2004). A novel NADPH thioredoxin reductase, localized in the chloroplast, which deficiency causes hypersensitivity to abiotic stress in *Arabidopsis thaliana*. *J. Biol. Chem.* 279 43821–43827 10.1074/jbc.M404696200 15292215

[B56] Shah J. Tsui F. Klessig D. F. (1997). Characterization of a salicylic acid-insensitive mutant (sai1) of *Arabidopsis thaliana*, identified in a selective screen utilizing the SA-inducible expression of the tms2 gene. *Mol. Plant Microbe Interact.* 10 69–78 10.1094/MPMI.1997.10.1.69 9002272

[B57] Shaikhali J. Heiber I. Seidel T. Stroher E. Hiltscher H. Birkmann S. (2008). The redox-sensitive transcription factor Rap2.4a controls nuclear expression of 2-Cys peroxiredoxin A and other chloroplast antioxidant enzymes. *BMC Plant Biol.* 8:48 10.1186/1471-2229-8-48 PMC2386467 18439303

[B58] Shaikhali J. Noren L. De Dios Barajas-Lopez J. Srivastava V. Konig J. Sauer U. H. (2012). Redox-mediated mechanisms regulate DNA binding activity of the G-group of basic region leucine zipper (bZIP) transcription factors in *Arabidopsis*. *J. Biol. Chem.* 287 27510–27525 10.1074/jbc.M112.361394 22718771 PMC3431687

[B59] Spoel S. H. Mou Z. Tada Y. Spivey N. W. Genschik P. Dong X. (2009). Proteasome-mediated turnover of the transcription coactivator NPR1 plays dual roles in regulating plant immunity. *Cell* 137 860–872 10.1016/j.cell.2009.03.038 19490895 PMC2704463

[B60] Srivastava V. Srivastava M. K. Chibani K. Nilsson R. Rouhier N. Melzer M. (2009). Alternative splicing studies of the reactive oxygen species gene network in Populus reveal two isoforms of high-isoelectric-point superoxide dismutase. *Plant Physiol.* 149 1848–1859 10.1104/pp.108.133371 19176719 PMC2663752

[B61] Stogios P. J. Downs G. S. Jauhal J. J. Nandra S. K. Prive G. G. (2005). Sequence and structural analysis of BTB domain proteins. *Genome Biol.* 6 R82 10.1186/gb-2005-6-10-r82 PMC1257465 16207353

[B62] Suzuki N. Koussevitzky S. Mittler R. Miller G. (2012). ROS and redox signalling in the response of plants to abiotic stress. *Plant Cell Environ.* 35 259–270 10.1111/j.1365-3040.2011.02336.x 21486305

[B63] Tada Y. Spoel S. H. Pajerowska-Mukhtar K. Mou Z. Song J. Wang C. (2008). Plant immunity requires conformational changes [corrected] of NPR1 via *S*-nitrosylation and thioredoxins. *Science* 321 952–956 10.1126/science.1156970 18635760 PMC3833675

[B64] Toone W. M. Morgan B. A. Jones N. (2001). Redox control of AP-1-like factors in yeast and beyond. *Oncogene* 20 2336–2346 10.1038/sj.onc.1204384 11402331

[B65] Tron A. E. Bertoncini C. W. Chan R. L. Gonzalez D. H. (2002). Redox regulation of plant homeodomain transcription factors. *J. Biol. Chem.* 277 34800–34807 10.1074/jbc.M203297200 12093803

[B66] Wang D. Amornsiripanitch N. Dong X. (2006). A genomic approach to identify regulatory nodes in the transcriptional network of systemic acquired resistance in plants. *PLoS Pathog.* 2:e123 10.1371/journal.ppat.0020123 PMC1635530 17096590

[B67] Wulff R. P. Lundqvist J. Rutsdottir G. Hansson A. Stenbaek A. Elmlund D. (2011). The activity of barley NADPH-dependent thioredoxin reductase C is independent of the oligomeric state of the protein: tetrameric structure determined by cryo-electron microscopy. *Biochemistry* 50 3713–3723 10.1021/bi200058a 21456578

[B68] Zheng M. Aslund F. Storz G. (1998). Activation of the OxyR transcription factor by reversible disulfide bond formation. *Science* 279 1718–1721 10.1126/science.279.5357.1718 9497290

